# MIMO Antennas: Design Approaches, Techniques and Applications

**DOI:** 10.3390/s22207813

**Published:** 2022-10-14

**Authors:** Preeti Sharma, Rakesh N. Tiwari, Prabhakar Singh, Pradeep Kumar, Binod K. Kanaujia

**Affiliations:** 1 Physics Division, School of Basic and Applied Sciences, Galgotias University, Greater Noida 203201, India; 2 Department of Applied Sciences, Greater Noida Institute of Technology, Greater Noida 201310, India; 3 Department of Electronics & Communication Engineering, Madanapalle Institute of Technology & Science, Madanapalle 517325, India; 4 Discipline of Electrical, Electronic and Computer Engineering, University of KwaZulu-Natal, Durban 4041, South Africa; 5 School of Computational and Integrative Sciences, Jawaharlal Nehru University, New Delhi 110067, India; 6 Department of Electronics and Communication Engineering, Dr. B. R. Ambedkar National Institute of Technology, Jalandhar 144011, India

**Keywords:** MIMO antennas, dual-band, circularly polarized MIMO antennas, isolation techniques, diversity parameters, 5G technology, 6G technology

## Abstract

The excessive use of digital platforms with rapidly increasing users in the wireless domain enforces communication systems to provide information with high data rates, high reliability and strong transmission connection quality. Wireless systems with single antenna elements are not able to accomplish the desired needs. Therefore, multiple-input multiple-output (MIMO) antennas are getting more attention in modern high-speed communication systems and play an essential part in the current generation of wireless technology. However, along with their ability to significantly increase channel capacity, it is a challenge to achieve an optimal isolation in a compact size for fifth-generation (5G) terminals. Portable devices, automobiles, handheld gadgets, smart phones, wireless sensors, radio frequency identification and other applications use MIMO antenna systems. In this review paper, the fundamentals of MIMO antennas, the performance parameters of MIMO antennas, and different design approaches and methodologies are discussed to realize the three most commonly used MIMO antennas, i.e., ultra-wideband (UWB), dual-band and circularly polarized antennas. The recent MIMO antenna design approaches with UWB, dual band and circularly polarized characteristics are compared in terms of their isolation techniques, gain, efficiency, envelope correlation coefficient (ECC) and channel capacity loss (CCL). This paper is very helpful to design suitable MIMO antennas applicable in UWB systems, satellite communication systems, GSM, Bluetooth, WiMAX, WLAN and many more. The issues with MIMO antenna systems in the indoor environment along with possible solutions to improve their performance are discussed. The paper also focuses on the applications of MIMO characteristics for future sixth-generation (6G) technology.

## 1. Introduction

Due to the usage of internet platforms in a variety of areas, wireless systems with high data rates and adequate channel capacity are in great demand. These requirements are usually incompatible with single-input and single-output (SISO) antennas. As a result, multiple-input and multiple-output (MIMO) printed antennas, a new form of antenna design, has emerged as a suitable candidate for high-speed communication technologies [1,2]. In such designs, two or more radiating elements are fed separately using a coplanar or strip line feeding technique to transmit and receive the data. However, the coupling between the ports is a major concern in MIMO design because it degrades the performance of MIMO antennas. As a result, several attempts have been undertaken to increase the isolation between the radiators. One of the ways to achieve good isolation in MIMO antennas is to use a metamaterial-based MIMO design [3,4]. For better decoupling, a suspended meta-surface made up of periodic square split-ring resonators (SRRs) are placed above the antenna array [5]. A split ring comprising an inductive line and a capacitive gap to achieve magnetic and electric coupling, respectively, is used to build the decoupling structure [6]. The orthogonally structured MIMO antenna provides excellent isolation [7,8]. In MIMO systems, latency can be reduced by massive MIMO configurations [9]. Rectangular strip based decoupling structure is used for isolation improvements [10]. A well-designed defective ground structure (DGS) along with the decoupling conducting strip printed between the radiating elements decreases the mutual coupling [11]. The theory of characteristic modes is also used for MIMO antenna systems in order to build a systematic technique to predict the isolation improvement [12]. With the advent of ultra-compact circuits, the isolation between each terminal of a MIMO system becomes crucial in order to achieve high system accuracy [13,14].

In this review paper, MIMO antenna designs and their characteristics are reported in three different categories. This paper primarily focuses on well-organized information about MIMO antenna design approaches with specific methodologies in order to achieve the desired antenna performance. Moreover, the comparison tables provided in the manuscript will help readers to implement and modify the described techniques for better performance of MIMO antennas. This paper can provide better insight for the possible directions of research that can be carried out in the future including THz technology for upcoming 6G communication systems. This manuscript is organized in eight sections, starting with the introduction; in Section 2, the basic categories of MIMO antenna design are presented; in Section 3, different MIMO designs with adopted techniques to achieve ultra-wideband characteristics are described. Further, in Section 4 and Section 5, dual-band and circularly polarized MIMO antennas with corresponding design methodologies are presented. In Section 6, the effect of antenna performance under the indoor region is discussed followed by the MIMO antenna design in the THz region for 6G applications in Section 7. Finally, the review work is concluded in Section 8. The reported MIMO antenna designs cover various applications such as WLAN, Bluetooth, 5G/6G, millimeter-wave and other high-speed communication systems.

## 2. MIMO Antenna Design Approaches

MIMO antennas have received much attention in the modern wireless communication system as it can use multi-paths to transmit or receive data, and hence increase the range and output performance [15]. It is noted that the significant isolation between components of the same MIMO system is necessary so that elements of the MIMO antenna can work independently to transmit or receive signals simultaneously without deteriorating the antenna parameters.

In order to ensure the quality of a MIMO antenna, in addition to S-parameters and radiation characteristics, certain diversity parameters are used. The MIMO antennas must satisfy the predefined values of the diversity parameters for practical applications. Therefore, some basic diversity parameters for MIMO antennas are mentioned in this section.

### 2.1. Envelope Correlation Coefficient (ECC)

The envelope correlation coefficient (ECC) is the diversity parameter which indicates the correlation between the adjacent MIMO antenna elements. It can be calculated from the radiation patterns or S-parameters. However, the ECC value evaluated using the far field radiation pattern is highly preferred because the ECC states how multiple radiating elements in MIMO systems are independent in their radiation pattern. Moreover, it is seen that most of the planar antennas suffer from loss; therefore, the method for determining ECC using the S-parameters should be avoided. The mathematical expression for ECC using the radiation pattern data of MIMO design is given by Equation (1) [11]:

(1) $${\mathrm{ECC}}_{qp}=\frac{{\left|\int_{0}^{2\pi }\int_{0}^{\pi }\left(E_{\theta p}^{*}E_{\theta q}P_{\theta }XPR+E_{\varphi p}^{*}E_{\varphi q}P_{\varphi }\right)d\Omega \right|}^{2}}{\alpha \times \beta }\;\;$$

here,

$$\alpha =\overset{2\pi }{\underset{0}{\int }}\overset{\pi }{\underset{0}{\int }}\left(E_{\theta q}^{*}E_{\theta q}P_{\theta }XPR+E_{\varphi q}^{*}E_{\varphi q}P_{\varphi }\right)d\Omega$$

$$\beta =\overset{2\pi }{\underset{0}{\int }}\overset{\pi }{\underset{0}{\int }}\left(E_{\theta p}^{*}E_{\theta p}P_{\theta }XPR+E_{\varphi p}^{*}E_{\varphi p}P_{\varphi }\right)d\Omega$$

where XPR is the cross-polarization level defined as the ratio of average power along the phi and theta directions. In a practical environment, the acceptable limit of ECC must be <0.5.

### 2.2. Diversity Gain (DG)

Diversity gain indicates the quality and reliability of a MIMO antenna in wireless systems. Hence, the DG of the MIMO antenna must be high (≈10 dB) within the acceptable frequency band. The DG is calculated using the ECC value and can be given by Equation (2) [4,11]:

(2) $$\mathrm{DG}=10\times \sqrt{1-{\left|{\mathrm{ECC}}_{qp}\right|}^{2}}$$

### 2.3. Channel Capacity Loss (CCL)

The CCL is defined as the maximum limit up to which the information can be transmitted with almost zero loss in the communication channel. The predefined CCL value for a given MIMO system is <0.4 bits/s/Hz.

The expression of CCL using S-parameters is given by Equation (3) [14]:

(3) $$\mathrm{CCL}=-log_{2}det\left(\vartheta^{\mu }\right)$$

where

$$\vartheta^{\mu }=\left[\begin{array}{cc} \xi_{11} & \xi_{12}\\ \xi_{21} & \xi_{22} \end{array}\right]$$

and

$$\begin{array}{cc} \xi_{11}= & 1-\left[{\left|S_{11}\right|}^{2}+{\left|S_{12}\right|}^{2}\right]\\ \xi_{12}= & -\left[S_{11}^{*}S_{12}+S_{21}^{*}S_{12}\right]\\ \xi_{21}= & -\left[S_{22}^{*}S_{21}+S_{12}^{*}S_{21}\right]\\ \xi_{22}= & 1-\left[{\left|S_{22}\right|}^{2}+{\left|S_{21}\right|}^{2}\right] \end{array}$$

### 2.4. Mean Effective Gain (MEG)

MEG is an important diversity parameter for MIMO antennas and defined as the ratio of power received by the MIMO antenna to the power received by the isotropic antenna. For the better performance of a MIMO antenna with the equal power level, the ratio MEGjMEGi must be less than 3 dB. The MEG can be evaluated using Equations (4) and (5) [14]:

(4) $${\mathrm{MEG}}_{i}=0.5\left[1-{\left|S_{ii}\right|}^{2}-{\left|S_{ij}\right|}^{2}\right]$$

and

(5) $${\mathrm{MEG}}_{j}=0.5\left[1-{\left|S_{ij}\right|}^{2}-{\left|S_{jj}\right|}^{2}\right]\;\;$$

### 2.5. Total Active Reflection Coefficient (TARC)

The TARC of the MIMO system is defined as the ratio of the total reflected power from the radiating elements to the total incident power on the patch. The expression for the generalized TARC for an N-port MIMO antenna is given by Equation (6) [10]:

(6) $$\mathrm{TARC}=\frac{\sqrt{\sum_{i=1}^{N}{\left|b_{i}\right|}^{2}}}{\sqrt{\sum_{i=1}^{N}{\left|a_{i}\right|}^{2}}}$$

Here, $b_{i}=\left[s\right]\cdot a_{i}$; [s], [b] and [a] are the scattering matrix, the scattering vector and the excitation vector, respectively.

Further, for two-port MIMO antennas, TARC can be given by Equation (7):

(7) $$\mathrm{TARC}=\frac{\sqrt{\left({\left|\left(S_{11}+S_{12}e^{j\theta }\right)\right|}^{2}+{\left|\left(S_{21}+S_{22}e^{j\theta }\right)\right|}^{2}\right)}}{\sqrt{2}}$$

The TARC is the ratio of the reflected power and the incdent power, and it is expected that all the power should be accepted by the antenna. Therefore, ideally the TARC value for the MIMO antenna should be zero. While designing the MIMO antenna, one has to optimize the values of all the antenna diversity parameters within the aformentioned predefined limit.

**Figure 1:**
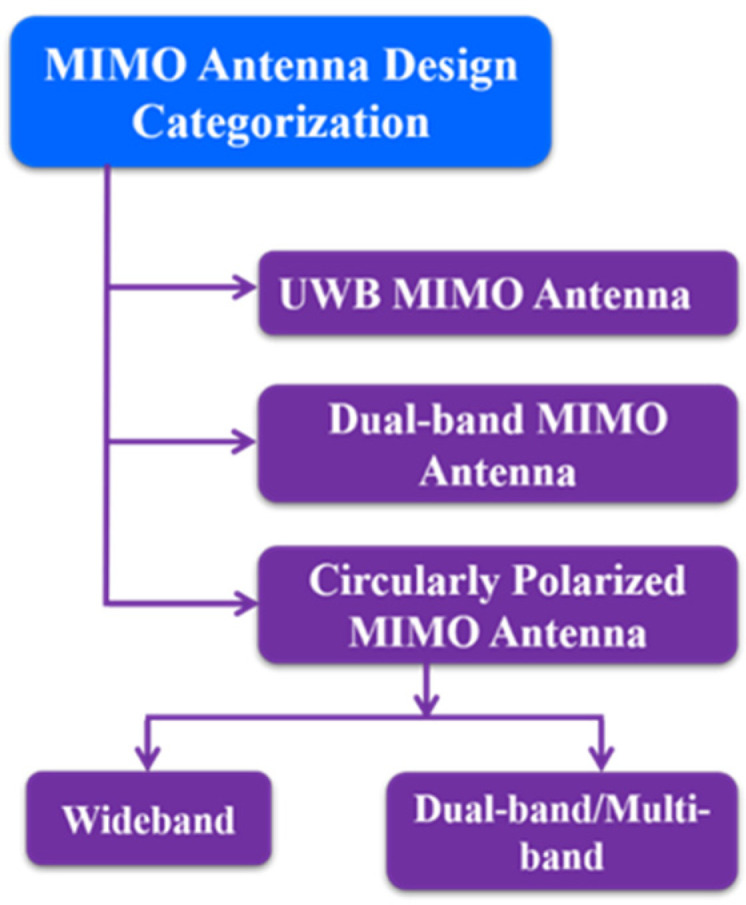
Categorization of MIMO antenna designs.

In this review paper, the MIMO designs are categorized based on the number of elements used and the type of characteristics demonstrated by the antenna. Most of the MIMO antennas available in the literature belong to UWB, dual band or circularly polarized types and are classified in Figure 1. The details of each type of MIMO design are further described in the following sections.

## 3. Ultra-Wideband (UWB) MIMO Antenna Designs

**Figure 2:**
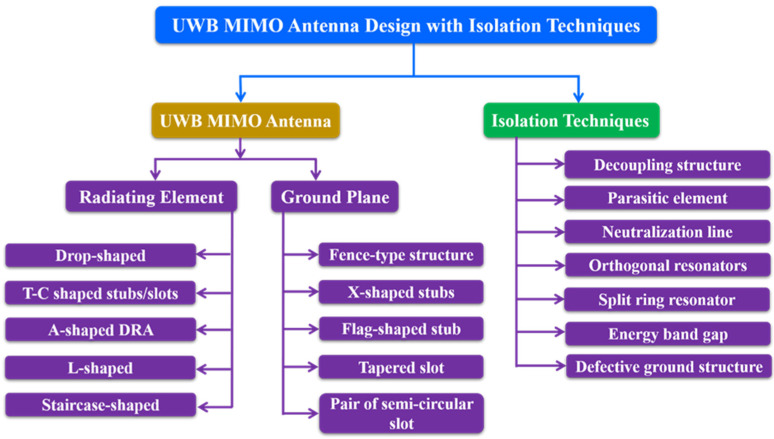
Typical UWB MIMO antenna design approaches.

Ultra-wideband (UWB or digital pulse wireless) is a radio-based wireless communication technology that allows the transmission of a huge amount of digital data over a broad range of frequency bands with extremely little power over short distances. In this section, the different UWB MIMO antennas are reported which includes modification in the radiating patches, ground plane (GP) and isolation techniques (Figure 2). UWB technology offers a frequency range of 3.1 to 10.6 GHz with channel bandwidths of more than 500 MHz [16]. For the decoupling of a small UWB MIMO antenna, a broadband neutralized line is employed [17]. With a mutual coupling of less than −22 dB, this UWB MIMO antenna has covered the lower UWB range of 3.1–5 GHz. MIMO designs must display wideband features to meet the demands of increased spectral efficiency, provided that the reciprocal coupling between the radiating components is kept to a minimum [17,18,19]. The decoupling network using two inverters and two connected split-ring resonators (SRRs) in [20] provides excellent isolation between the ports.

**Figure 3:**
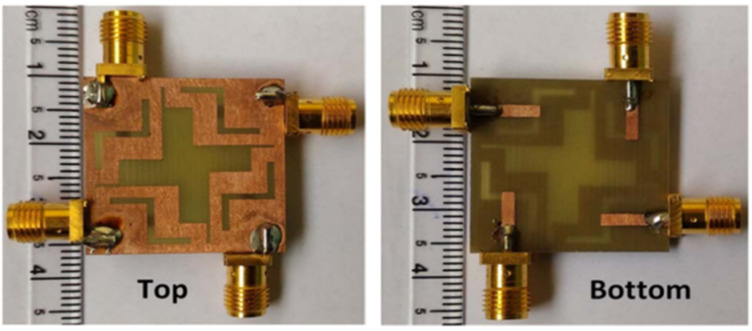
The fabricated prototype of 4 × 4 MIMO antenna [21]. (**Left side**: ground plane and **right side**: microstrip line).

**Figure 4:**
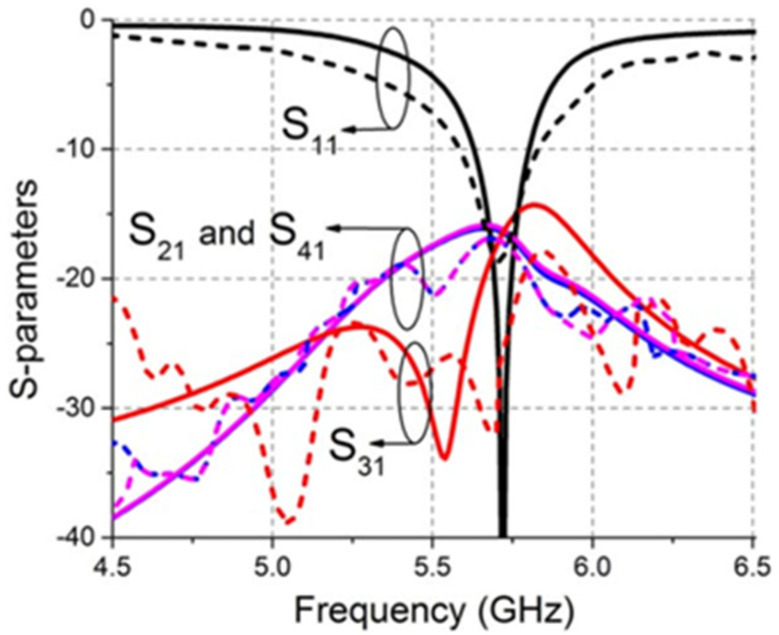
S–parameters of the MIMO antenna. — simulated and --- measured [21].

A four-element compact MIMO antenna with a total volume of 26 × 26 × 0.8 mm^3^ and a 4 mm edge-to-edge distance between the radiating patches is reported in [21]. A groove is carved in the center of the GP (Figure 3) to prevent mutual coupling. The MIMO antenna functions with isolation of more than 15.4 dB in the frequency region from 5.6 to 5.8 GHz (Figure 4). The maximum achieved gain for this antenna is >1.41 dBi across the applicable band and can be implemented in portable devices for WLAN applications at 5.7 GHz.

**Figure 5:**
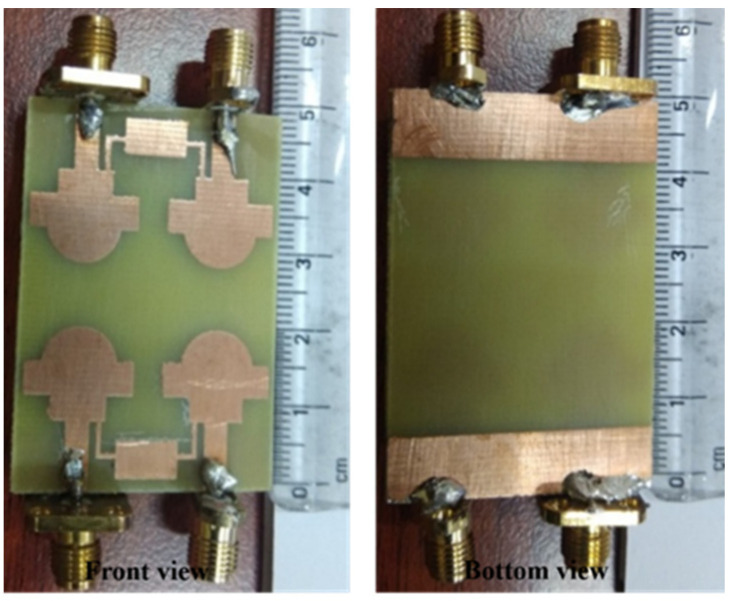
Fabricated 4 × 4 MIMO antenna design [22].

**Figure 6:**
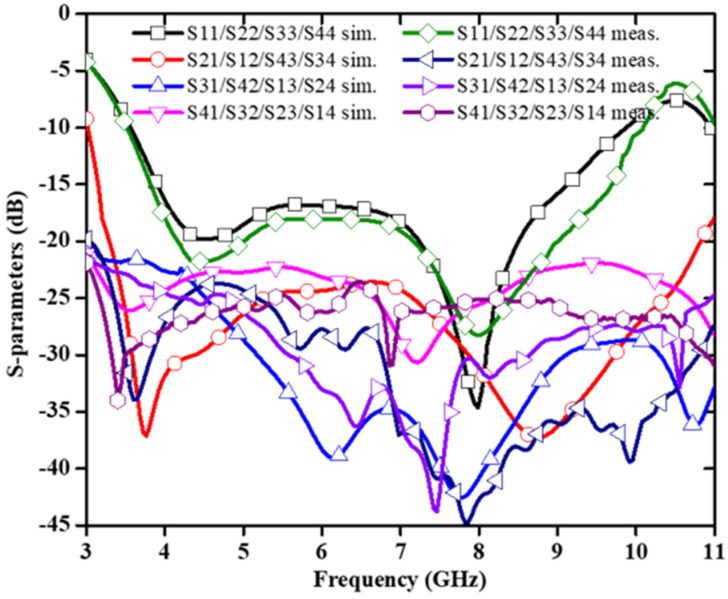
S–parameters of the designed antenna [22].

Figure 5 shows the structure of the 4 × 4 UWB MIMO antenna as reported in [22]. The dimensions of the reported antenna is 48 × 34 × 1.6 mm^3^. The MIMO design consists of the rectangular patch attached with a half-circular disc of radius 4.8 mm. The lower corners of the rectangular patch are carved with a small amount of copper to achieve UWB characteristics. The neutralization line connects the two symmetrical antennas to increase the isolation between the two radiating patches. The corresponding S-parameters are plotted and presented in Figure 6. The observed bandwidth and isolation of 2 × 2 and 4 × 4 MIMO antennas are 95.22% (3.51–9.89 GHz) and −24 dB, and 96.47% (3.52–10.08 GHz) and −23 dB, respectively.

**Figure 7:**
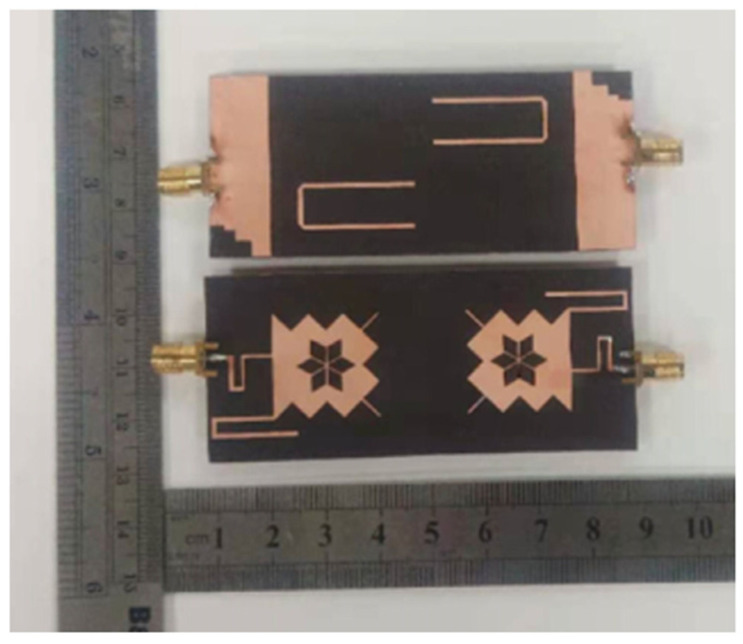
Fabricated prototype of the structure [23].

**Figure 8:**
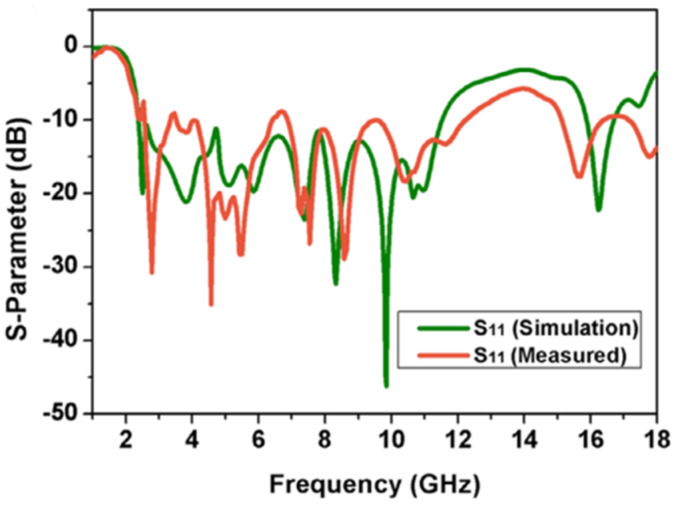
Simulated and measured S–parameters of the antenna [23].

In [23], a UWB MIMO antenna with defective ground structure (DGS), U-shaped branch step impedance stub, and slotted patch with dimensions of 80 × 35 × 0.508 mm^3^ is reported and shown in Figure 7. The DGS improves the antenna bandwidth significantly. By adding U-shaped branches, the antenna achieves different resonance characteristics. Step impedance converters produce better impedance matching as compared to standard linear transmission lines. The diagonal arrangement of U-strips is employed to increase the isolation of the antenna. The antenna gain ranges from 3.58 to 5.79 dBi, and from Figure 8 it is found that the relative impedance bandwidth of the design is 131% (2.57–12.2 GHz). The maximum measured value of isolation is −15 dB with an ECC value < 0.005.

A corner-truncated rhombus-shaped radiating patch is responsible for UWB range [24]. Moreover, to enhance the decoupling effect, a small rectangular stub is connected in the GP and placed between two ports. The MEG is measured using a far-field radiation pattern, which shows a value of −6 dB. A compact wideband MIMO antenna has two identical monopole radiating components engraved on top of the substrate along with a GP including a flag-shaped stub and strips at the bottom [25]. UWB frequency is achieved by adding the slits and slots situated next to the flag-shaped stub. This antenna supports wireless systems such as LTE, WiMAX, WLAN, and HIPERLAN and runs on several standards. In [26], the neutralization line is used between the radiating elements to cancel the field between the neighboring radiating components. The antenna impedance changes as a result of the field cancellation and the coupling between the antennas is decreased. To enhance impedance bandwidth, a UWB MIMO antenna implements two symmetric slot antenna components with quasi F-shaped radiators and L-shaped open-slots [27]. The currents flowing between the closely spaced patches are ejected through a tapered slot carved on the array GP, resulting in a novel antenna with a decoupling structure as suggested in [28]. The idea adopted in this design is to separate coupled currents by passing them through a tapered slot. In [29], the wideband operation is achieved by a central conjoined circular slot. In [30], a UWB MIMO design achieves better isolation without the need of any decoupling techniques taking full use of the monopole asymmetrical structure. In this design, wide bandwidth is achieved by using a radiating patch with a split form. To increase the isolation, two elements on the front side of the MIMO antenna are placed orthogonal to the back-side elements and a circular radiating patch provides good wideband operation [31]. In addition to that, decoupling structures are placed on the top and bottom layers of the substrate to further reduce the mutual coupling between the ports. To decrease mutual coupling, spatial diversity and polarization diversity techniques are applied and a staircase-shaped patch provides UWB frequency range [32]. In [33], four drop-shaped slot antennas are placed perpendicular to one another but in separate layers to limit the transmission of the surface current to the other radiators for improving the isolation. Seven vertical metallic barriers are placed on the front side of the antenna and two L-shaped stubs along with a vertical metallic barrier printed on the back side to strengthen the isolation between the two radiating elements [34]. In addition to that, a circular radiating patch with a small hemisphere on the top of the circle is utilized to achieve wider bandwidth. The introduction of a fence-type decoupling structure at the GP improves the isolation in the working band and the rectangular slot on the radiation patch enhances the resonance characteristics [35]. Moreover, two L-shaped parasitic branches are added on the surface of the dielectric substrate with the goal of improving the mutual coupling at lower frequency (3–3.4 GHz) and the impedance matching performance. In [36], a MIMO antenna is made up of two identical antenna elements that are symmetrically arranged in opposite directions on the same dielectric substrate. High isolation and good impedance matching are accomplished by loading three X-shaped stubs between two disconnected ground planes. In [37], the antenna is made up of four similar patches arranged in an orthogonal pattern. The total size of the antenna is reduced by using a rectangular GP below the radiating patch to produce a monopole design. The use of T- and C-shaped stubs/slots on the radiating patch reduces interfering bands for WiMAX, LTE43, and WLAN and achieve super wideband [38]. Dielectric resonator antennas (DRA) [39,40,41] can also be used to develop a dual-element wide band MIMO antenna system. The wide bandwidth is achieved by merging the two neighboring modes of a wideband A-shaped DRA stimulated by a simple rectangular conformal strip [39]. A mushroom-shaped dielectric resonator activated by a conformal trapezoidal patch is reported in [40] to achieve wideband operation.

The various reported UWB MIMO antennas along with their bandwidth enhancement, decoupling techniques and diversity parameters are presented in Table 1.

## 4. Dual-Band MIMO Antenna Designs

**Figure 9:**
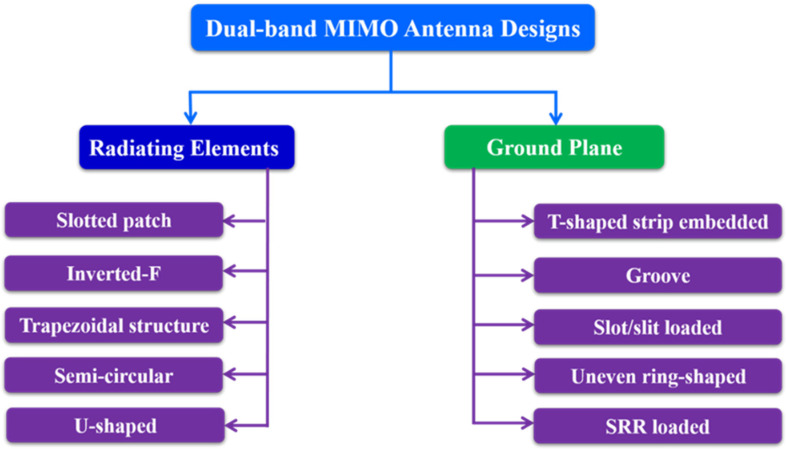
Dual-band MIMO antenna design approaches.

A dual-band antenna transmits and receives radio signals at two different frequencies. These antennas may use any of the two frequencies separately or both at the same time, depending on their arrangement and applications. Dual-band antennas are widely used with 2.4 GHz and 5 GHz frequency bands. Dual-band allows for quicker speeds and greater versatility. As a result, the dual-band eliminates connection problems and provides more reliability, flexibility and stability. The high gain and omnidirectional Wi-Fi coverage of dual-band antennas [42] make them perfect for a wide range of applications, including vast interior areas, warehouses, buildings, naval installations and many more. Figure 9 shows a design approach for dual band MIMO antennas. The dual-band MIMO antenna designed for WLAN application is made up of closely spaced symmetric MIMO antennas with a 5.3 mm edge-to-edge spacing [43].

Additionally, a decoupling network is added between the two patches to improve isolation without increasing the footprint. The dual band characteristics are achieved by changing the length of two monopole arms. An elliptical slot and a rectangular parasitic strip are used to establish isolation over the operating dual bands [44]. A dual-band MIMO antenna with two C- shaped patches is employed and a bent T-shaped structure is implanted between the two monopole radiators to provide good isolation [45]. To decrease inter-element coupling, an open slit in the GP is used in a dual-band MIMO antenna design [46].

**Figure 10:**
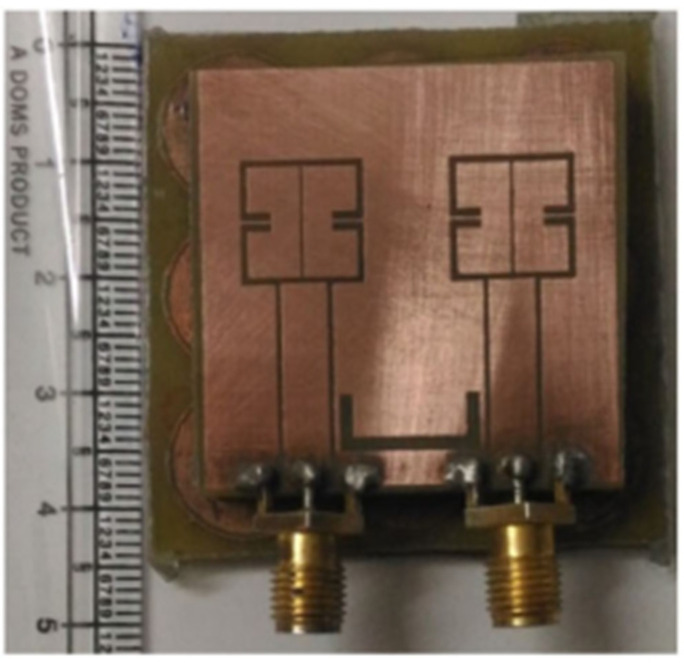
Prototype of CPW-fed MIMO design [47].

**Figure 11:**
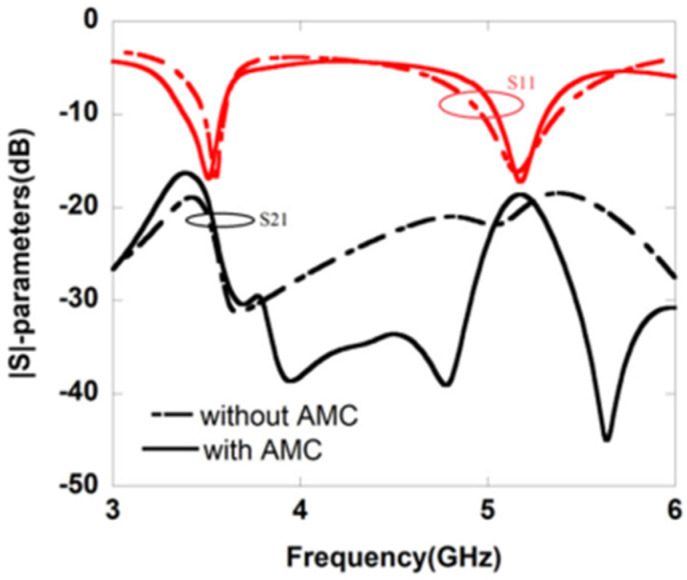
Simulated S–parameters for MIMO antenna [47].

**Figure 12:**
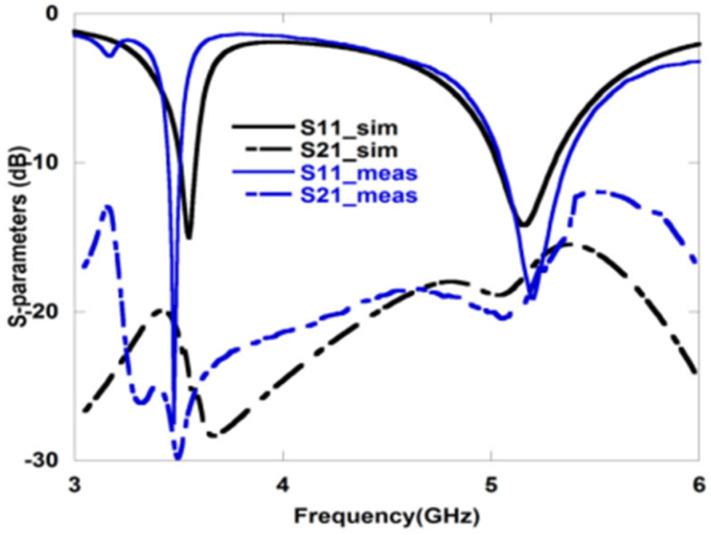
Measured S–parameters for dual-band MIMO antenna [47].

In [47], back-to-back C-type slot resonators are connected in a radiating patch of a CPW-fed dual-band MIMO antenna using an artificial magnetic conductor as shown in Figure 10. The antenna elements are separated by a gap of 8.3 mm, and the antenna resonates at 3.5 and 5.2 GHz (Figure 11 and Figure 12). Although, there is already a significant amount of isolation at the lower band, a U-shaped slot is introduced in the GP to further boost the isolation at the upper frequency band.

**Figure 13:**
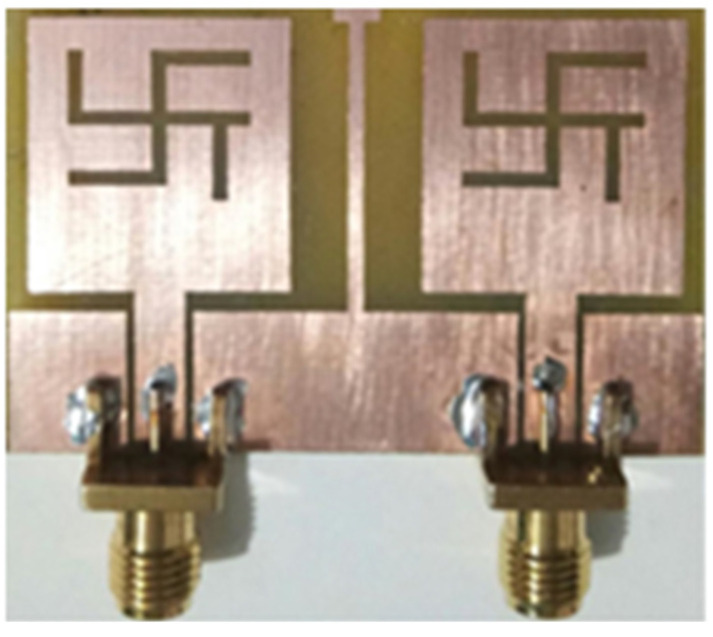
Fabricated two-element dual-band MIMO antenna [48].

A 2 × 2 dual-band MIMO antenna with size of 46 × 30 × 1.6 mm^3^ is reported in [48]. A swastika-shaped slot in the rectangular patch makes up the intended dual-band CPW-fed MIMO antenna as shown in Figure 13. Moreover, to increase the isolation between the two radiating components, a T-shaped narrow conducting strip is connected in the GP.

**Figure 14:**
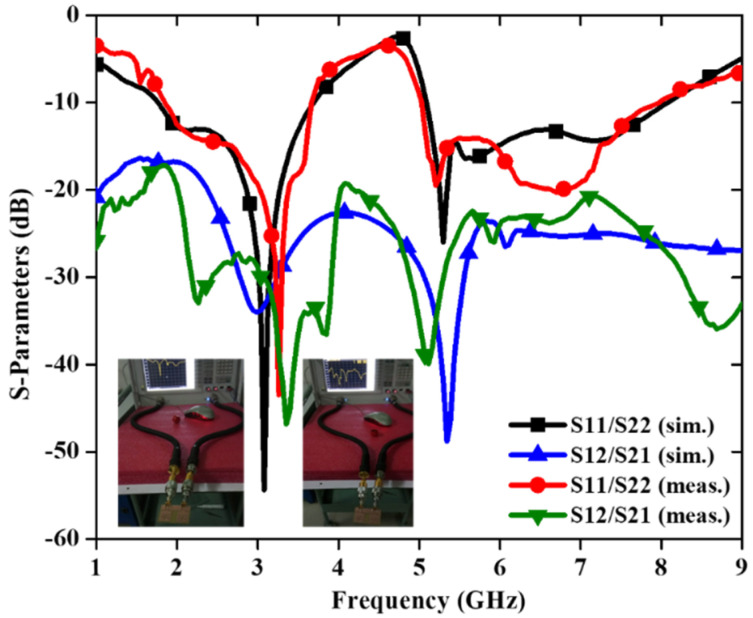
S–parameter of the dual-band MIMO antenna [48].

It is found that lower and higher frequency bandwidths of the design are 64.96% (1.85–3.63 GHz) and 44.36% (5.07–7.96 GHz), with isolation values ≤−17.21 dB and ≤−22.42 dB, respectively (Figure 14). The observed realized gain varies between 1.14 and 4.12 dBi (lower band) and 1.42 and 4.78 dBi (upper band), with a radiation efficiency > 72% for both frequency bands. This MIMO antenna covers the applications in DCS, LTE2300/2500, Bluetooth, ISM, and WLAN. The radiation patterns at 3.12, 5.29 and 7.18 GHz are evaluated and it is found that the E-plane pattern is dumb-bell shaped while the H-plane is omnidirectional in nature. The T-shaped strip embedded in the ground plane minimizes the electromagnetic coupling between the patches and hence the radiation patterns are least affected.

**Figure 15:**
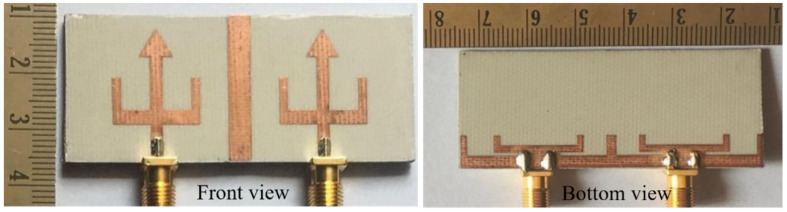
Top and bottom view of fabricated antenna [49].

In [49], a trident-shaped dual-port MIMO antenna with dimensions 62 × 25.6 × 1.524 mm^3^ is shown in Figure 15.

**Figure 16:**
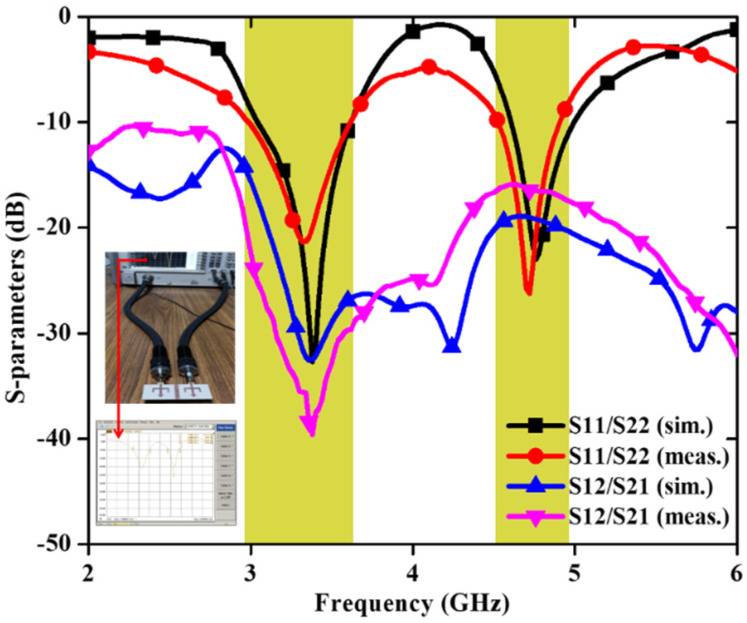
S–parameters of dual-band MIMO antenna [49].

To obtain the dual-band characteristics, an arrow-shaped strip is placed between the U-shaped patch with two L-shaped slots on the GP. The dual frequency bands cover the frequency range from 2.99 to 3.61 GHz (lower band) and 4.53 to 4.92 GHz (higher band) with isolation ≤ −25 dB and ≤−16 dB, respectively (Figure 16). This MIMO antenna effectively covers the 5G and sub 6G n77/n78/n79 spectrum.

The directional dual-band planar inverted-F antenna (PIFA) element with a small dimension of 31 × 17 mm^2^ is presented in [50], in which two antenna elements share a common ground with a 3.0 mm edge-to-edge inter-element spacing. Further, it is found that the inverted L-shaped metal arm dimensions are responsible for dual band operation. An inverted T-Shape slot is created on the GP to produce lower and higher frequency bands [51]. A T-shaped strip and a rectangular strip make up the radiating element in [52] and the lower and higher frequency bands are primarily matched by the top and bottom half of the T-shaped strip and the rectangular strip. In [53], six rectangular slots on the trapezoidal structure patch produce two frequency bands and they are achieved by adjusting the length of the rectangular slots. In addition to that, a T-shaped branch contributes to the improvement in isolation of the designed MIMO antenna. In [54], a MIMO antenna with decoupling structure is reported consisting of four L-shaped branches arranged in a counterclockwise direction. Dual band response of the antenna is achieved by varying the length and width of the L-shaped branches. In [55], inverted L-shaped monopole antennas loaded with split-ring resonators (SRRs) are placed in a rotationally symmetric pattern. The inverted L-antenna is responsible for the higher band and by the interaction of the inverted L-antenna and interconnected GP, the lower frequency band is created. The SRR loading makes it easier to build a wide-band antenna mode that can span across lower WLAN, WiFi, and WiMAX application bands. In [56], a meander dipole, a concave parabolic reflector, and a parasitic strip are used to provide good impedance matching in the antenna. The parabolic reflector is used to reduce antenna size and also to improve the lower band directivity. The upper band is created using a metal strip. To accomplish the dual band antenna, in [57], the radiating patch has two asymmetric U-shaped slots printed on the substrate. Improved isolation is achieved by using a composite GP with four metallic strips. In [58], to produce a tiny dual-band WLAN MIMO antenna, lower and higher band dummy elements are employed. The desired frequency bands are achieved by adjusting the width of two branches and the angle between the branches. In [59], to produce a dual band, a rectangle split-ring-resonator (RSRR) slot is placed into the patch. Two arrays of 2 × 1 antenna elements make up an optically transparent MIMO antenna in which a slotted circular ring monopole antenna with a partial GP is used to increase the operation bandwidth and dual band operation [60]. A 2 × 2 MIMO antenna is presented in [61] using a semi-annular patch embedded with a zig-zag conducting strip. It is seen that the higher frequency band is controlled by the zig-zag structure while the lower band is unchanged. Further, the isolation is improved using a fork-shaped feedline. In [62], for a dual-band MIMO antenna, the mushroom type EBG is placed between two antenna elements, providing excellent isolation and reducing mutual coupling. In [63], four simple elliptical-shaped patches are arranged orthogonally around a plus-shaped partial GP. Two opposite slots are introduced into the patch elements to achieve dual band and the plus shape improves the isolation of the designed MIMO antenna. Two symmetrical monopole antenna elements make up the reported MIMO antenna in [64]. By modifying the current distribution on the GP, a DGS and grounded branches are loaded to minimize mutual coupling across the lower band. Between the two antenna elements, there is a T-shaped parasitic element which introduces an inverted path for mutual coupling cancellation, considerably improving the isolation at higher frequency band. As a result, this antenna is capable for 5G dual-band operation. In [65,66], the slots are created on the GP to achieve dual-band MIMO antennas. In [67], for WiMAX/WLAN applications, a compact dual-band MIMO-stacked DRA is designed. The total height of the antenna is 7.0 mm, and the ground area is 50 × 50 mm^2^. The DGS approach is employed to create strong isolation between antenna ports.

The various dual-band MIMO antenna designs and the different antenna parameters are compared in Table 2. This comparison is presented in terms of antenna size, MIMO elements, and various techniques to achieve the dual-band operation and better isolation.

## 5. Circularly Polarized MIMO Antenna Design Approaches

**Figure 17:**
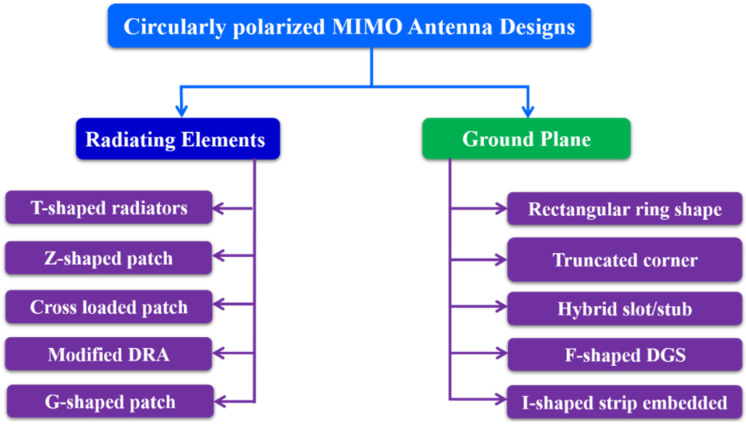
Circularly polarized MIMO antennas design approaches.

Circularly polarized (CP) antennas have several significant advantages over linearly polarized antennas. In long-distance communication, circular polarization reduces the losses carried on by polarization mismatch. Additionally, circularly polarized antennas will reduce the spread of multipath propagation delays [68]. CP antennas are rapidly becoming a key component for a wide range of wireless systems, including satellite communications, mobile communications, wireless sensors, radio frequency identification (RFID), wireless power transmission, WLAN [69], wireless personal area networks (WPAN) and global navigation satellite systems (GNSS) [70]. Figure 17 shows some typical design approaches of circularly polarized MIMO antennas. Moreover, some latest designs and methodologies to realize CP MIMO antennas are described in this section.

**Figure 18:**
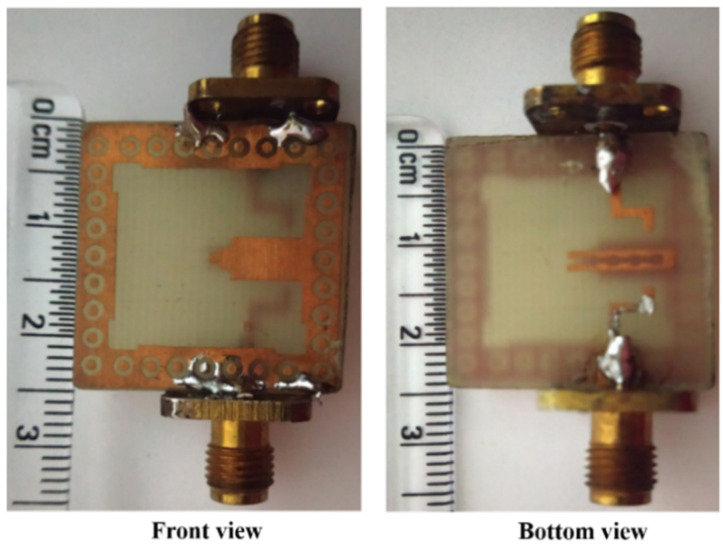
Prototype of circularly polarized MIMO antenna [71].

**Figure 19:**
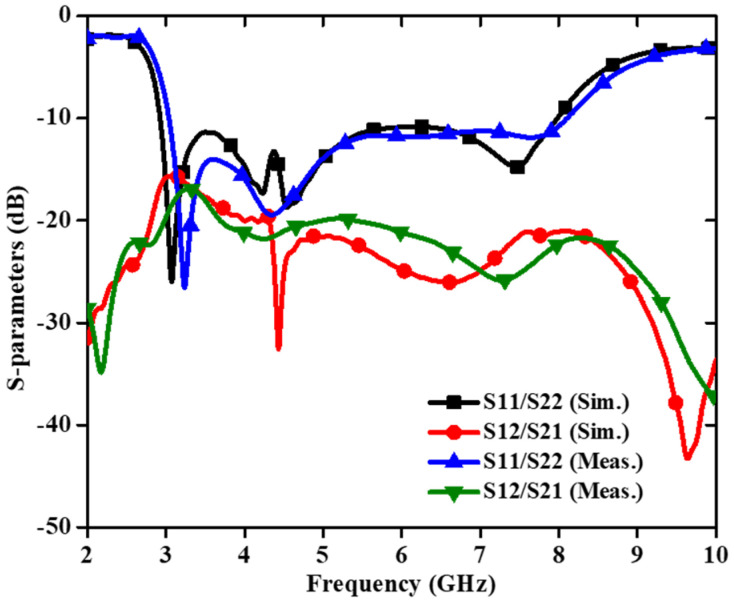
S–parameters of circularly polarized MIMO antenna [71].

**Figure 20:**
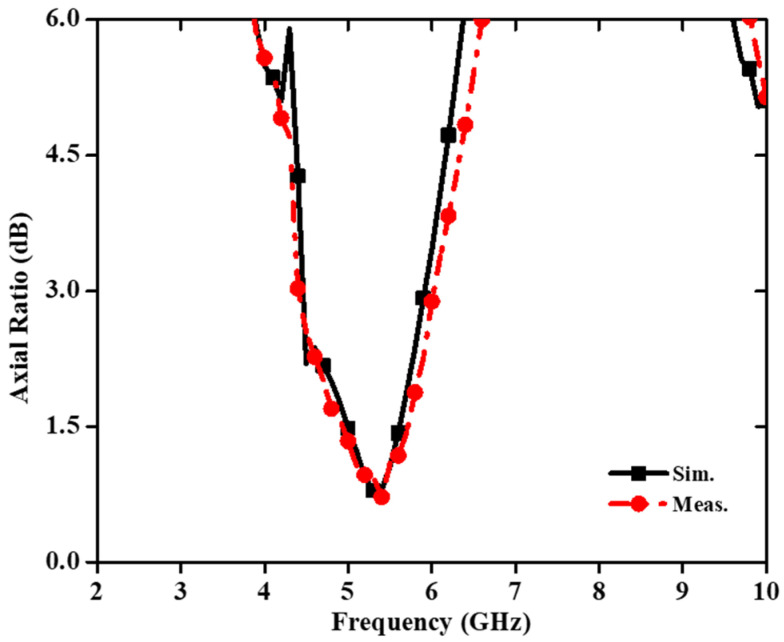
Axial ratio of CP-based MIMO antenna [71].

Figure 18 shows a small and compact two-port circularly polarized MIMO antenna with dimensions 24 × 24 × 1.6 mm^3^ [71]. The circularly polarized radiated field of the MIMO antenna is achieved using a unique ground structure implanted with rectangular slots and Z-shaped radiating patches. A meandering U-shaped narrow metallic strip, which provides the good isolation, is placed between the asymmetric Z-shaped radiating components. The impedance bandwidth (3.04–8.11 GHz) of this antenna is 90.94% (Figure 19) and a 3 dB axial ratio bandwidth of 32.10% (4.42–6.11 GHz) is achieved (Figure 20). The ECC and CCL of the reported antenna are found to be 0.004 and 0.32, respectively.

**Figure 21:**
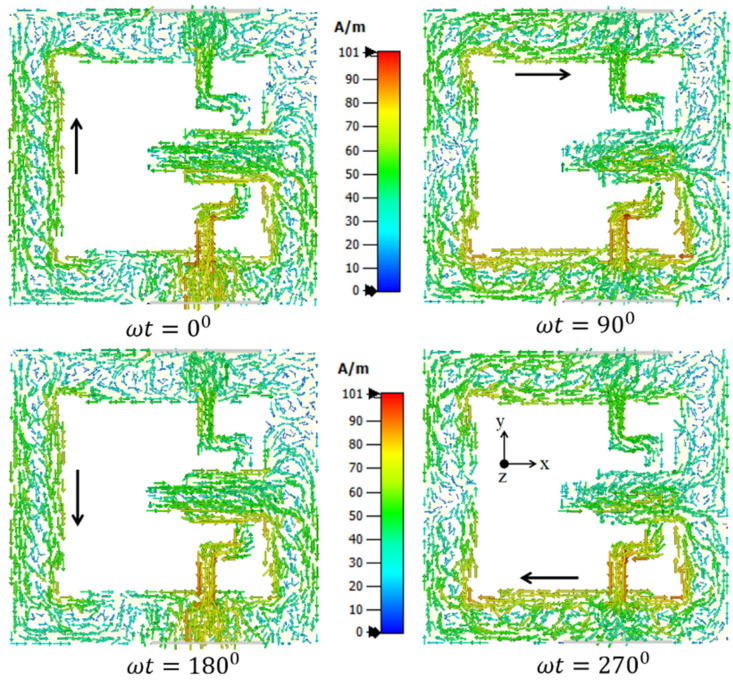
Surface current density at 5.45 GHz [71].

**Figure 22:**
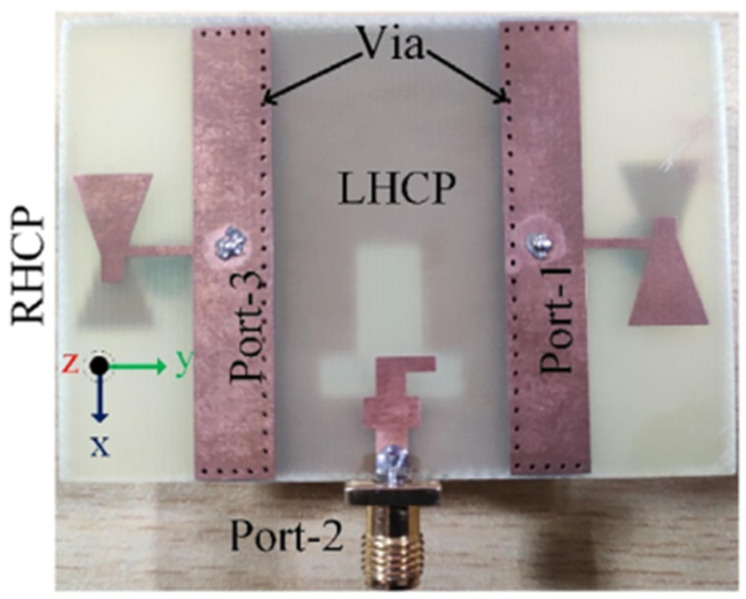
Fabricated circularly polarized MIMO antenna [72].

The circular polarization mechanism can be explained by using the current distribution graph shown in Figure 21. From the figure it is seen that the current density is largely concentrated along the y-axis at time instants t = 0°. The current direction on the radiating patch and on the GP are in opposing directions at t = 90°, but the current flowing on the GP along the x-axis is maximum, and therefore the radiation occurs. With regard to time phase, the present rotation is clockwise, and the radiation is left-hand circular polarization (LHCP) in the +z direction. Furthermore, when the current is rotated in the −z direction, the current rotates anticlockwise, and the radiation received is right-hand circular polarization (RHCP). The radiation pattern is symmetrical, and the RHCP is 18 dB greater than the LHCP in the boresight direction. As a result, there is excellent cross-polarization rejection. In [72], a broadside circularly polarized T-shaped slot antenna and two end-fire CP antennas are combined to create a three-port MIMO antenna system for WLAN application (5.15–5.35 GHz). Figure 22 shows a T-shaped slot antenna with L-shaped feed line to excite the CP wave in the broadside direction. The parallel arrangement of an electric dipole and magnetic dipole with a 90° phase shift is used to create the end-fire CP antenna.

**Figure 23:**
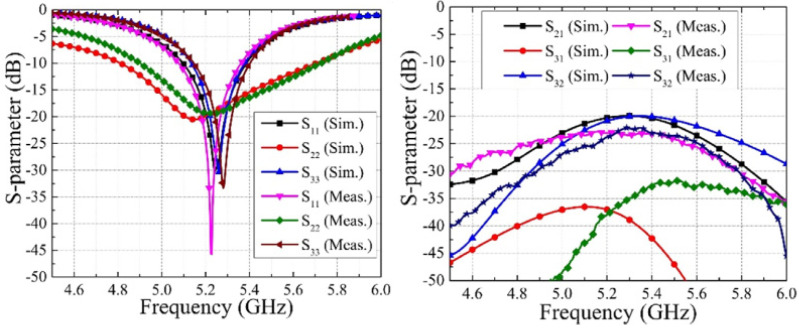
S–parameters of three-port CP MIMO antenna [72].

**Figure 24:**
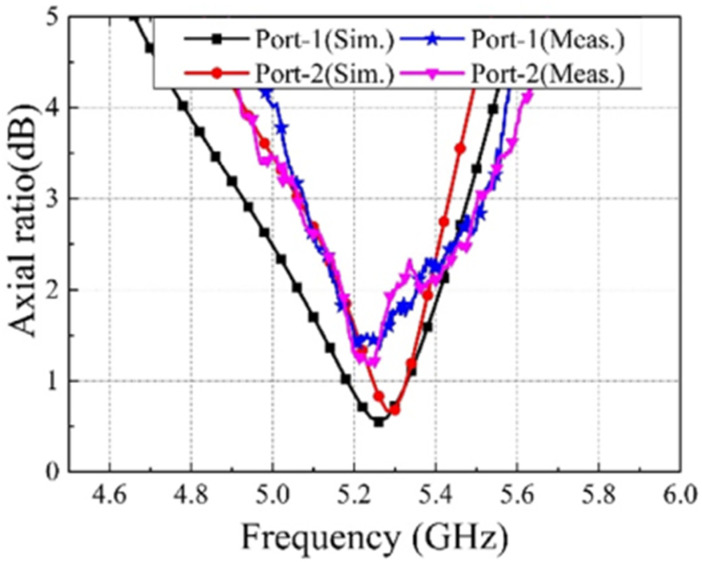
Simulated and measured axial ratio of CP MIMO antenna [72].

**Figure 25:**
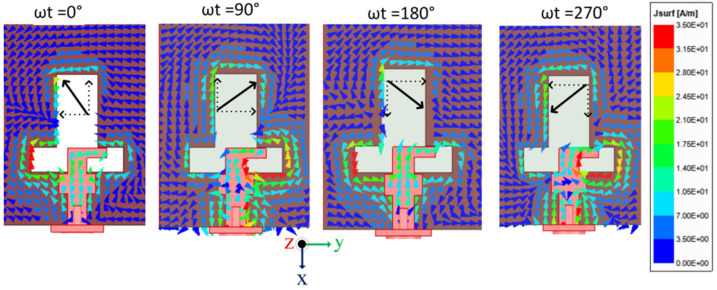
Surface current distribution in 3-port MIMO antenna [72].

Figure 23 and Figure 24 show the S-parameters and axial ratio of the designed CP MIMO antenna. Figure 25 shows that a horizontally polarized wave in the +y direction is produced at ωt = 0° by the electric dipole. While the electric field distribution along the open-ended cavity is robust at time phase ωt = 90°, the current distribution on the electric dipole is small. As a result, at ωt = 90°, a vertically polarized wave along the +y-direction is excited by the magnetic dipole. The generation of CP waves along the +y-direction is caused by this 90° phase difference between the electric and magnetic dipoles. Similar effects are also seen for time phases at ωt = 180° and ωt = 270° [72].

In [73], the MIMO antenna uses an array of periodic metallic plates and two square patches with distinct truncated corners for polarization variety. The antenna emits RHCP and LHCP waves when corners of the patches are truncated properly. DRA proves to be a better alternative in terms of CP and impedance bandwidth [74,75,76,77,78,79,80,81]. To achieve wideband AR performance, 45° partial truncations at two opposing corners of the dielectric resonator are paired with an optimal ratio cross-ring coupling slot. To connect the feeding structure, glue is used to attach the DRA to the PCB. Small triangular supports are added to the bottom of the DR to allow it to be mounted on the PCB without causing any problems [74]. In [75], two cylindrical dielectric resonators and two symmetrical modified Y-shaped microstrip-printed lines are used to create a MIMO antenna. Two orthogonal modes are generated using a Y-shaped printed line. As a result of the feeding arrangement, CP waves are generated. To improve the isolation, two metallic strips and a slit on the GP are employed. In [76], as a consequence of the MIMO antenna orientation, CP radiation is achieved. To make the structure simple for obtaining CP radiation, an L-shaped DR is employed. The antenna components are 19 mm apart when placed side by side. In [77], a pair of self-complementary slot-based DGS is utilized to reduce mutual coupling between the antenna elements, and L-shaped DR is used to achieve CP radiation with the help of a common coaxial probe feeding network. In [78], by integrating the parasitic patch and incorporating the diagonal location of DRAs, isolation between the radiators is improved. Because of the proper implementation of the parasitic patch, CP is attained. In [82], the edge-to-edge spacing between the two patches of MIMO design is 12.2 mm for the best results. The basic square patch with square cut at the corner provides circular polarization. Moreover, impedance matching, high isolation and better AR bandwidth are achieved by creating a slot in the ground plane. In [83], to achieve high gain and desired impedance bandwidth, the MIMO design includes four CP pentagonal microstrip components with a suspended substrate of 6 mm height and five loaded slits. The center-to-center distance of the elements is taken as 0.655λ_0_. In [84], a circularly polarized MIMO antenna having four G-shaped components is reported in which two demonstrate LHCP and other two show RHCP. In the G-shaped element, a vertical line strip achieves 90° phase change for circular polarization and the I-shaped strip on the GP achieves an identical voltage level. In [85], for high isolation, three grounded stubs and a mirrored F-shaped DGS are used, and circular polarization is achieved using simple offset feeding. In [86,87], the usage of a ring-shaped DRA provides the increased bandwidth. By converting rectangular to Z-shaped slots, circular polarization is obtained in both working bands. Two diagonal slotted square patches are designed in [88] to improve the bandwidth. Furthermore, to eliminate mutual coupling, four parasitic elements are used. In [89], a broadband 2 × 1 CP array antenna with a mutual coupling of less than −25 dB and a 0.3 edge-to-edge spacing is reported and a parasitic line patch is added to the array to improve the isolation between the radiating elements. The line patch width is increased to expand AR Bandwidth. In [90], a MIMO antenna is reported consisting of two port T-shaped radiators and a rectangular-shaped GP which helps to create circular polarization. The LHCP wave is generated by the clockwise rotation of surface currents on the T-shape antenna radiator and rectangular-shaped GP at different phase angles. In both E- and H-planes, the isolation between the LHCP and RHCP patterns is >15 dB, demonstrating that the developed antenna possesses strong cross-polarization discrimination. In [91], a modified square patch serves as the radiating structure. The truncated corner approach is used to obtain the CP, and an additional open-circuited stub on the GP is used to increase the CP bandwidth. Two orthogonal modes with the same amplitude are produced by utilizing the hook-shaped open-circuited stub and truncated corner patch. 

The various circularly polarized MIMO antenna designs and the different antenna parameters along with diversity performance are compared in Table 3. The different methods used to obtain CP characteristics and AR bandwidth are presented in the table.

## 6. MIMO Antennas in Indoor Environment

MIMO antennas, when installed within the inhouse region, suffer with the major issue of reduced channel capacity. Some efforts have been made to improve the capacity performance of MIMO systems. Applying frequency-selective (F-S) wallpaper to the walls to block undesired interference with the desired radio communication services is a potential method of resolving indoor situations [92,93]. Considering both SISO and MIMO systems, the features and design of the new F-S wallpaper are reported in [94]. The wallpaper, which is built on symmetric and periodic metallic hexagons, is designed and applied to the ordinary walls in order to block 5 GHz transmissions without obstructing the other radio communication services. The wallpaper is created using the periodic boundary finite-difference time-domain (PB-FDTD) approach based on the unit cell analysis method. In [95], interference levels are decreased using the wallpaper inside a 4 × 4 MIMO system for indoor environments. This MIMO design consists of an array of half-wavelength dipoles for the receiving antenna and a cavity-backed dynamic meta-surface antenna (DMA) for the transmission of the signal. While the transmitter’s location is fixed, the MIMO channels are simulated and the channel capacity is calculated at various receiver locations using the ray tracing approach. Compared to a MIMO system using a sub-aperture phased array for the transmitting antenna, the reported adaptive radiation pattern provided by the DMA can achieve excellent capacity improvement [95].

## 7. MIMO Characteristics for 6G Technology

Although the 5G mobile communications standard is still in the growing stage of deployment, investigations are in pace for the next generation of wireless technology, 6G wireless systems. In order to overcome the operational difficulties experienced by fifth-generation cellular technology, 6G communication systems are designed to accommodate growing data-hungry applications with boosting connectivity and enhanced network capabilities [96]. The bandwidth and latency of 6G networks will be significantly higher than those of 5G networks due to their ability to operate at higher frequencies. One of the objectives of the 6G internet is to provide communications with a latency of even less than microseconds. It is predicted that 6G would provide extremely dependable low-latency communication with a strong emphasis on internet devices, the application of artificial intelligence in wireless communication and the improvement of mobile broadband [97]. Massive MIMO technology is currently used in 5G communication networks; on the other hand, there will be a need for dozens or even hundreds of antennas and radio links at the base station when 6G technology will be deployed [98]. At the same time, due to the large scale of deployment, there will be high hardware costs, power usage and complicated designs [98]. To fulfill the requirement, upcoming communication systems (6G) are moving towards the higher frequency bands, such as the terahertz (THz) and millimeter-wave bands [99,100]. The internet of nano-things, health monitoring systems, entertainment services, military and ultra-high-speed on-chip communications are some of the significant uses of THz band wireless communication [97,100]. This has encouraged researchers to continuously develop current wireless networks in order to switch over to 6G cellular systems.

## 8. Conclusions

A comprehensive study on design approaches and applications of MIMO antennas was presented in this paper. Design approaches for ultra-wideband MIMO antennas, dual-band MIMO antennas and circularly polarized MIMO antennas were discussed. The presented study is very useful for researchers working in the field of MIMO antennas. From this study, it is found that the UWB characteristics of MIMO antennas can be achieved by modifying the radiating patches as L-shaped, staircase-shaped, and square-shaped embedded with slits and slots and use of stubs in the patch. Furthermore, creating defects in the GP which includes fence-shaped GPs, different stub loaded designs and tapered slots/slits are some of the methods to improve the antenna bandwidth. Dual-band MIMO antennas can be obtained by incorporating the slots in the radiating patches, U-shaped patches and modification of patches to resonate in the dual/multi frequency band. It is also observed that the uses of SRR-loaded and groove-loaded GP also provide dual- band MIMO antennas. CP-based MIMO antennas are designed by simultaneously optimizing the structure of the patch and the GP. Some basic radiating structures such as cross-loaded patches, G-shaped and Z-shaped patches, and square and circular patches embedded with slots at specific positions and some modified DRAs are used for CP characteristics. The truncated corners and slots itched in the GP with a specific shape, and use of hybrid slots within and at the periphery of the GP are common techniques to improve the CP quality in the MIMO antenna. In addition to that, the isolation techniques in each type of MIMO design are described and presented in tables. In most of the MIMO designs, decoupling parasitic lines are used between the ports to improve the isolation. Moreover, the neutralization line, SRR and EBG structures in the GP as well as between the radiating elements are used for better decoupling. Placing the radiating elements more than the half wavelength of the designed antenna minimizes mutual coupling. Orthogonal orientation of the resonators is also utilized for mutual coupling reduction. The comparison of various antenna parameters including the size, isolation techniques, design methodologies and other characteristics provide an overview of the specific MIMO antenna design. Thus, it is concluded that this review paper will surely help a lot to enhance the quality and performance of MIMO antenna designs which are demanded by the high data transmission rates of present communication systems, as well as the upcoming 6G technology.

## Figures and Tables

**Table 1:** Comparison of different UWB MIMO antennas.

Ref.	MIMO Element	Antenna Size (mm^3^)	Antenna Frequency Band (GHz)	Bandwidth Improvement Technique	Isolation (dB) with Decoupling Techniques	Gain (dBi)	Efficiency (%)	ECC	CCL (bits/s/Hz)
[22]	4 × 4	48 × 34 × 1.6	3.52–10.08	Modified rectangular patch	≤−23 Neutralization line	0.95–2.91	70.01–79.87	≤0.039	≤0.29
[24]	2 × 2	25 × 36 × 1.6	2.78–17.43	Corner-truncated rhombus-shaped (CTRS)	<−19 Rectangular strip connected with GP	-	-	<0.008	<0.31
[25]	2 × 2	36 × 22 × 1.6	2.5–2.85/4.82–6.1	Square radiating patch	<−18 Flag-shaped stub connected with middle GP	9.992	-	<0.05	-
[26]	2 × 2	21 × 34 × 1.6	3.52–9.89	Dome-shaped patch	≤−22 Neutralization line	3.08–5.12	>62	≤0.005	<0.26
[27]	2 × 2	30 × 50 × 1	3–10.9	F-shaped radiators with L-shaped open-slots	≤−20 Fork-shaped slots	1.9–38	-	<0.06	-
[28]	2 × 2	22 × 43.5 × 1	2.45	L-shaped radiating patch	<−40 Tapered slot	1.9	81.7	0.06	-
[29]	4 × 4	42 × 42 × 1	3.3–4.2	Four conjoined slots	<−10 Circular slot	-	47–64	<0.06	-
[30]	4 × 4	45 × 45 × 1.6	4.3–6.45	Split-shaped radiating patch	<−20 Decoupling structure	4.0–5.0	90	<0.2	<0.018
[31]	4 × 4	40 × 40 × 1.6	3.1–11	Circular patch	<−20 Decoupling structure	3.28 (avg. gain)	-	<0.004	<0.4
[32]	3 × 3	45 × 25 × 1.588	3.1–11.5	Staircase-shaped radiators	≤−19 Spatial diversity	5.5 (peak gain)	61–98	≤ 0.2	-
[33]	4 × 4	110 × 110 × 1.45	1.7–7.2	Kraus technique	<−20 Electro-magnetic walls	3.0–5.2	90	0.0025	-
[34]	2 × 2	29.5 × 60 × 1.6	3.05–20	L-like stubs	<−20 Metallic barriers	3.36–4.92	83	<0.00012	0.325
[35]	2 × 2	50 × 35 × 1	3.0–11	L-shaped parasitic branches	<−25 Fence-type decoupling structure	above 3 dB	>80	<0.004	-
[36]	2 × 2	18 × 28 × 1.6	1.9–14	Three crossed X-shaped stubs	<−15.5 X-shaped stubs in ground planes	0.4–4.8	-	<0.09	<0.4
[37]	2 × 2	16 × 26 × 1.6	2.82–14.45	Circular radiator	<−22 Stubs and protruded strip	0.7–6.86	≥91.7	<0.08	-

**Table 2:** Comparison of different dual-band MIMO antennas.

Ref. No.	MIMO Element	Antenna Size (mm^3^)	Antenna Frequency Band (GHz)	Technique to Achieve Dual-band	Isolation (dB) with Decoupling TECHNIQUES	Gain (dBi)	Efficiency (%)	ECC	CCL (bits/s/Hz)
[42]	2 × 2	20 × 34 × 16	2.11–4.19/4.98–6.81	Embedding a pair of comb-shaped slots in the GP	<−21 T-stub with comb-shaped slots	2.75–4.19	>70	<0.004	<0.32
[48]	2 × 2	46 × 30 × 1.6	1.85–3.63/5.07–7.96	Swastika-shaped slot in the rectangular patch	<−17.21 T-shaped narrow conducting strip in GP	1.14–4.12/1.42–4.78	71.21–92.69/70.55–90.99	<0.003	<0.35
[49]	2 × 2	62 × 25.6 × 1.524	2.99–3.61/4.53–4.92	Arrow-shaped strip in between the U-shaped patch	<−16 Defected ground with L-shaped slot with strip	2.96–3.14/3.69–3.84	72.68–80.24	<0.002	<0.32
[50]	2 × 2	69 × 34 × 4.2	2.375–2.52/4.98–5.88	Inverted F-shaped	<−18 Slots on GP	2.66/5.18	-	<0.01	-
[51]	2 × 2	52 × 77.5 × 1.6	2.4–2.48/5.15–5.825	Horizontal U-strip	<−15 Inverted T-slot and meander line resonancebranch	-	-	<0.2	-
[52]	2 × 2	32 × 32 × 1.59	2.36–2.59/3.17–3.77	T-shaped strip and rectangular strip	<−15 Rectangular microstrip stub with defected GP	5.8 (peak gain)	76	<0.02	-
[53]	2 × 2	30 × 30 × 1.6	3.32–3.74/5.45–6.05	Trapezoidal-shaped patch	<−20 T-shaped branch	<1.5/3.5 (peak gain both band)	-	-	-
[54]	4 × 4	30 × 30 × 0.8	4.58–6.12	Rectangular patch	<−15.4 Swastika- shaped decoupling strip	4.02	67–82	<0.15	-
[55]	4 × 4	40 × 40 × 1.6	2.93/5.68	L-shaped with split ring resonator	<−14 SRR	4	83.48–89.55	<0.05	<0.5
[56]	4 × 4	85 × 85 × 0.8	2.32–2.95	Metal strip	<−14 Parasitic element	5.5	83 −90	<0.008	-
[57]	4 × 4	38 × 38 × 1.6	2.38–2.45/2.96–4.01	Two asymmetric U-shaped slots in the radiating patch	≤−18 Four metallic strips in the GP	-	-	<0.008	<0.35
[58]	2 × 2	70 × 70 × 0.8	2.4~2.5/5.6~5.8	Width of branches	<−25 Loadeddummy elements	-	Not given value	Not given value	-
[59]	2 × 2	72 × 56 × 0.8	2.24–2.90/3.9–7.55	Rectangle split-ring-resonator	<−24 ITI-shaped structure	2.5–5.6	-	<0.04	<0.4
[60]	2 × 2	105 × 105 × 1.83	2.23–2.46/3.22–4.04	Slotted interconnected ring resonator	<−12	3.6/7.1 (peak gain)	74–84	0.002	-
[62]	1 × 2	51 × 29.6 × 1.6	2.4/5.2	Slotted rectangular patch	<−25 EBG structure	2.2/3.8 (peak gain)	-	0.07	-
[63]	4 × 4	58 × 60 × 1.6	1.55–2.65/3.35–3.65	Two opposite slots in the radiating elements	<−10 Orthogonal plus-shaped partial ground	2.2/3.8	-	<0.08	<0.4
[64]	2 × 2	38.6 × 56.4 × 1.524	3.5/4.85	L-shaped branches	<−29 DGS and ground branches	2.45/4.56	-	<0.005	-

**Table 3:** Comparison of different circularly polarized MIMO antennas.

Ref.	MIMO Element	Antenna Size (mm^3^)	Frequency Band (GHz)	3-dB AR Bandwidth (GHz)	CP Technique	Gain (dBi)	Isolation (dB)	ECC	CCL bits/s/Hz
[71]	2 × 2	24 × 24 × 1.6	3.04–8.11	4.42–6.11	Asymmetric Z-shaped patch with stub loaded defected GP	0.28–2.76	<−16	<0.004	<0.32
[73]	2 × 2	56 × 32 × 3	5.10–5.85	5.10–5.85	Truncated corner patch with defected periodic GP	5.8	≤−20	-	-
[74]	2 × 2	95 × 49.7 × 1.6	3.15–3.93	3.3–3.8	Cross ring slot with DRA truncation	4.83	<−26	< 0.03	<0.10
[75]	2 × 2	50 × 70 × 1.6	2.21–3.13/3.40–3.92/5.30–6.10	5.62–5.86	Dual strips along with single slot in the GP	4.1	<−28	<0.15	<0.23
[77]	2 × 2	40 × 65 × 1.6	5.16–6.30	5.20–5.58	L-shaped DRA	4.011	22.284	<0.112	<0.338
[78]	2 × 2	350 × 350 × 26.1	3.50–4.95	3.58–4.40	Rectangular DRA with parasitic patch	6.2	<−28	<0.04	-
[82]	2 × 2	22.5 × 50 × 1.6	5.2–6.4	5.37–5.72	Square slot cut in the corner of the GP	6 (Peak gain)	<−20	0.001	-
[84]	4 × 4	70 × 68 × 1.6	4–13	4.2–8.5	Cross-shaped structure on ground	6.4 (Peak gain)	≤−18	<0.25	-
[85]	2 × 2	150 × 100 × 0.8	2.47–2.55	2.50–2.66	Offset feeding	6.1 (Peak gain)	≤−20	0.003	-
[86]	4 × 4	80 × 80 × 11.6	3.35–3.82/5.09–5.41	3.54–3.72/5.04–5.16	Z-shaped slots	5.0–6.8	<−18	<0.04	-
[87]	2 × 2	80 × 40 × 1.6	2.9–3.2/3.44–3.64/4.75–5.5	3.32–3.58/ 5.0–5.32	Z-shaped slots in the GP	2	≤−15	<0.2	-
[88]	2 × 2	-	4.75–5.9	5.1–5.8	Parasitic elements	7.5–8.2	≤−22	-	-

## Data Availability

Not applicable.
